# Knowledge, attitudes, and impact of COVID-19 pandemic among neurology patients in Jordan: a cross-sectional study

**DOI:** 10.1186/s41983-021-00354-9

**Published:** 2021-07-29

**Authors:** Mohammad Athamneh, Qais Sa’di, Belal Aldabbour, Yousef Khader, Waleed Batayha

**Affiliations:** 1 Department of Neuroscience, Faculty of Medicine, Yarmouk University, Irbid, Jordan; 2 Department of Neurology, Princess Basma Teaching Hospital, Irbid, Jordan; 3 Department of Neuroscience, Faculty of Medicine, Jordan University of Science and Technology (JUST), Irbid, Jordan; 4 Department of Public Health and Community Medicine, Jordan University of Science and Technology, Irbid, Jordan

**Keywords:** SARS-CoV-2, Epidemic, Jordan, Health Services, Disease control

## Abstract

**Background:**

The impacts of the COVID-19 pandemic on health services offered to patients with non-communicable diseases, including chronic neurological illnesses, are diverse and universal. We used a self-reported questionnaire to investigate these impacts on neurology patients in Jordan and assess their knowledge and attitudes towards the pandemic.

**Results:**

Most respondents had positive attitudes towards the COVID-19 pandemic, with 96% reporting they believed in the seriousness of the pandemic and adhered to prevention measures. Nearly 97% resorted to the internet and media outlets for medical information about the pandemic. About one in five clinic visitors had their appointments delayed due to interruption of health services. A similar portion of patients with MS, epilepsy, and migraine or tension headache reported medication interruptions during the pandemic. One in two patients reported new events or worsening illness since the start of the pandemic, and sleep disturbances were reported by nearly one in three patients who had epilepsy or headache.

**Conclusion:**

The COVID-19 pandemic’s impacts on patients with neurological illnesses in Jordan were deep and diverse. Meanwhile, the majority of surveyed neurology patients demonstrated a positive attitude towards the pandemic.

## Background

The impact of pandemics on healthcare systems is well-documented, particularly in countries with limited resources. Routine health services decreased by an estimated 18% during the 2014–2015 Ebola outbreak in West Africa, resulting in thousands of potentially preventable deaths [1]. Also, following the severe acute respiratory syndrome (SARS) outbreak in China, clinic and emergency room visits at a hospital in Taipei City dropped to 55% and 45%, respectively, in 2003 compared with the previous year [2]. A study in Qatar revealed that the overall utilization of primary health care services declined to 50% in April of 2020 during the surge of local Coronavirus disease of 2019 (COVID-19) spread [3]. In Spain, a negative effect was observed on up to 85% of healthcare quality standards in Catalonia in March and April of 2020 [4]. Also, in the USA, a CDC report found a substantial reduction in pediatric vaccine orders following the COVID-19 emergency declaration in March of 2020 [5].

A growing body of literature suggests that severe acute respiratory syndrome coronavirus 2 (SARS-CoV-2), causing COVID-19, has neurotropic characteristics [6–9]. Neurologists have other considerations regarding the impact of the virus on their patients. Many neurological diseases necessitate long-term and profound immune suppression [10]. In addition, patients with neuromuscular diseases represent a particularly vulnerable group to which the infection can be potentially fatal [11]. The most immediate and possibly the broadest short-term impact of the pandemic on neurology patients could be the limitations on accessibility to healthcare and medications, especially in communities with uncontrolled spread or as a byproduct of strict prevention measures.

In Jordan, the authorities have since March 17, 2020, imposed a number of local and nationwide curfews and lockdowns in a bid to curb local spread and prevent overwhelming the healthcare system [12]. Subsequently, many hospitals had to discontinue outpatient and clinic services, and elective procedures were postponed for weeks or months. Also, many patients faced difficulties refilling prescriptions and obtaining regular medications. On the other hand, an online survey of 5274 persons from Jordan found that approximately four out of every ten participants experienced quarantine-related anxiety [13], illustrating one aspect of the pandemic’s impact on the health of the Jordanian population. This study aimed to explore the attitude towards the COVID-19 pandemic and its impacts on the health of patients with neurological illnesses including multiple sclerosis (MS), epilepsy, and primary tension or migraine headache.

## Methods

This is a cross-sectional study that was conducted between November and December of 2020. Patients aged ≥ 18 years who presented with a neurological complaint at outpatient neurology clinics at the hospital affiliated with the second and last authors during the study period were invited to participate in this study. This hospital is a tertiary facility, and it is the main governmental hospital in the city. After obtaining ethical approval, a paper-based questionnaire was administered to patients visiting the hospital’s neurology clinics. Written consent was obtained from each patient prior to the administration of the questionnaire. The questionnaire included questions about the attitude of patients towards the pandemic and the impact they feel it had on their lives and illnesses. Specific questions were included in the questionnaire relevant to those with an established diagnosis of MS, epilepsy, or primary tension or migraine headaches. Additionally, as sleeping disturbances can exacerbate epilepsy and headache, patients with these two conditions were asked about the occurrence of sleep disturbances during the pandemic and the predisposing factors. Patients were excluded if they presented with non-neurological complaints. Additionally, patients with advanced dementia or severe intellectual disability were excluded if their conditions precluded meaningful communication or affected their ability to answer the questionnaire independently and reliably. Statistical analysis of the collected data was conducted using IBM SPSS software version 25 (SPSS Inc., Chicago, IL, USA). Descriptive statistics were performed for all variables to calculate frequencies and percentages.

## Results

A total of 562 patients presented to neurology outpatient clinics during the study period, of whom 506 (90.03%) patients responded to the questionnaire. Patients under 40 years of age constituted over half the sample. Men constituted 45.45% of the sample. Five patients only (0.98%) reported a previous documented infection with COVID-19. The majority of patients (81.42%) presented to the clinic for follow-up, while 18.57% stated that this was their first visit. One fifth (19.36%) of patients reported that their visit was delayed due to the pandemic and the lockdowns. Most patients (88.14%) believed in the existence of COVID-19 virus and adhered to preventive measures, and 79.84% agreed that lockdowns were necessary to control the spread of the pandemic among the population. Only 1.97% of patients obtained pandemic-related information from doctors and medical sources, while the vast majority were depending on the internet and media to seek necessary information. Table 1 illustrates the characteristics of the study sample.

**Table 1:** Patients’ demographic characteristics and their perceptions of COVID-19

Variables	Multiple sclerosis (***n*** = 80) ***n*** (%)	Epilepsy (***n*** = 150) ***n*** (%)	Headache (***n*** = 40) ***n*** (%)	Total (***n*** = 506) ***n*** (%)
**Gender**				
Male	21 (26.25)	77 (51.33)	7 (17.50)	230 (45.45)
Female	59 (73.75)	73 (48.67)	33 (82.50)	276 (54.55)
**Age group**				
18–40	62 (77.50)	105 (70.00)	0 (00.00)	276 (54.55)
41–60	17 (21.25)	40 (26.67)	26 (65.00)	159 (31.42)
> 60	1 (1.25)	5 (3.33)	14 (35.00)	71 (14.03)
**Previous diagnosis with COVID-19**	1 (1.25)	3 (2.01)	1 (2.50)	5 (0.99)
**Visit to the clinic**				
First visit	4 (5.00)	8 (5.33)	12 (30.00)	94 (18.58)
Follow-up	76 (95.00)	142 (94.67)	28 (70.00)	412 (81.42)
**Appointments delayed due to the pandemic**				
Yes	31 (38.75)	32 (21.48)	11 (27.50)	98 (19.44)
No	49 (61.25)	117 (78.52)	29 (72.50)	406 (80.56)
**Belief in the existence of the virus and adherence to prevention measures**				
Neither belief nor adherence	3 (3.75)	7 (4.70)	1 (2.50)	11 (4.09)
Belief but without adherence	1 (1.25)	10 (6.71)	0 (0.00)	11 (4.09)
Belief and adherence	72 (90.00)	131 (87.92)	37 (92.50)	240 (89.22)
Belief and obsessive adherence impacting life	4 (5.00)	1 (0.67)	2 (5.00)	7 (2.60)
**Agreement with effectiveness of the lockdown**				
Effective	74 (92.50)	107 (71.81)	36 (90.00)	217 (80.67)
Ineffective	6 (7.50)	42 (28.19)	4 (10.00)	52 (19.33)
**Source of information regarding the pandemic**				
Internet and social media	57 (71.25)	72 (48.32)	24 (60.00)	153 (56.88)
Medical specialists	1 (1.25)	2 (1.34)	2 (5.00)	5 (1.86)
Radio and TV	21 (26.25)	71 (47.65)	13 (32.50)	105 (39.03)
No interest in information	1 (1.25)	4 (2.68)	1 (2.50)	6 (2.23)

Variability in totals is due to missing values in some variables

Among the 506 participants, 80 (15.81%) had MS. About 73.75% of patients with MS were females and 77.50% were 40 years old or younger. Thirty-five patients (43.75%) reported experiencing at least one MS relapse since the start of the pandemic. Of those, 30 (88.24%) were admitted to the hospital, while three (8.82%) patients have rejected admission for concerns regarding the COVID-19 pandemic, and one (2.94%) did not seek medical advice for non-pandemic related reasons. Seventy-one (88.75%) patients with MS had been on a disease-modifying treatment (DMT), while nine (11.25%) were not receiving any treatment due to either medical or financial reasons. Beta-interferon (*n* = 35, 43.75%) and Fingolimod (*n* = 30, 37.50%) were the most frequently used DMTs, followed by Dimethyl Fumarate (*n*=4, 5.00%), and Natalizumab (*n* = 2, 2.50%). Nine (11.25%) patients with MS were not taking any medication. Regarding adherence to DMT during the pandemic, 13 (16.25%) patients stated that they had discontinued the DMT during the pandemic, with four (30.76%) of them stating that noncompliance was related to concerns over the immunosuppressive side effects, and three (23.07%) reporting inability to obtain the medication due to the lockdown (Table 2).

**Table 2:** Health challenges among MS, epilepsy, and headache patients during COVID-19

	Multiple sclerosis (***n*** = 80) ***n*** (%)		Epilepsy (***n*** = 150) ***n*** (%)		Headache (***n*** = 40) ***n*** (%)	
Variables	Total	Patients with new relapses (%)	Total	Patients with increased seizure (%)	Total	Patients with increased headache (%)
**Discontinuation of medication (or regular infusions)**						
Yes	13 (17.57)	5 (38.46)	30 (20.13)	23 (76.67)	10 (25.00)	7 (70.00)
No	61 (82.43)	26 (42.62)	119 (79.87)	56 (47.06)	30 (75.00)	18 (60.00)
**Reason of discontinuation**						
Anxiety about the pandemic (and/or concerns regarding immune suppression side effects of DMT)	4 (30.77)	2 (50.00)	9 (34.62)	7 (77.78)	2 (20.00)	1 (50.00)
Restricted accessibility due to the pandemic	3 (23.08)	1 (33.33)	12 (46.15)	9 (75.00)	2 (20.00)	1 (50.00)
Others	6 (46.15)	2 (33.33)	5 (19.23)	3 (60.00)	6 (60.00)	5 (83.33)
**Change in sleep pattern**	–	–				
Fewer hours at night and during the day			16 (10.74)	13 (81.25)	9 (22.50)	8 (88.89)
Fewer hours at night and more during the day			13 (8.72)	6 (46.15)	3 (7.50)	1 (33.33)
More hours of sleep			12 (8.05)	5 (41.67)	3 (7.50)	0 (0.00)
No change			108 (72.48)	55 (50.93)	25 (62.50)	16 (64.00)
**Reason of change in sleep pattern**	–	–				
Anxiety and fear of the pandemic			14 (34.15)	11 (78.57)	4 (28.57)	3 (75.00)
Frequent lockdowns and work stoppage			15 (36.59)	5 (33.33)	3 (21.43)	0 (0.00)
Others^a^			12 (29.27)	8 (66.67)	7 (50.00)	5 (71.43)

Variability in totals is due to missing values in some variables

^a^“No specific reason” for epilepsy patients or “Headache itself” for headache patients

A total of 150 patients had epilepsy, accounting for 29.64% of the study sample. Most patients with epilepsy were younger than 40 (70.00%). Males and females constituted 51.33% and 48.66% respectively. Of this sample, eighty patients (53.33%) reported an increased frequency of seizures since the start of the pandemic. Interruptions of anti-seizure medication (ASM) intake was reported by thirty patients (20%), of whom 76.66% had increased seizures. In addition, sleep disturbances were reported by over a quarter of patients with epilepsy, and the majority ascribed these disturbances to the impact of the pandemic (Table 2).

In addition, 40 patients had primary migraine or tension-type headache, of which 37 (92.50%) patients had migraine headache, and three (7.50%) had tension headache. Females represented 82.22% of patients in this group, and 65% of all patients were younger than 40. An increased frequency of headache since the start of the pandemic was reported by 25 (62.50%) of patients in this group, and 25.00% reported interruption of their regular medication during the same period. Changes in sleep patterns were reported by 37.50% of patients in this group, and 50.00% blamed these changes to the impact of the pandemic. Of note, eight out of nine (88.8%) patients with decreased total sleep hours experienced worsening headaches (Table 2).

**Fig. 1:**
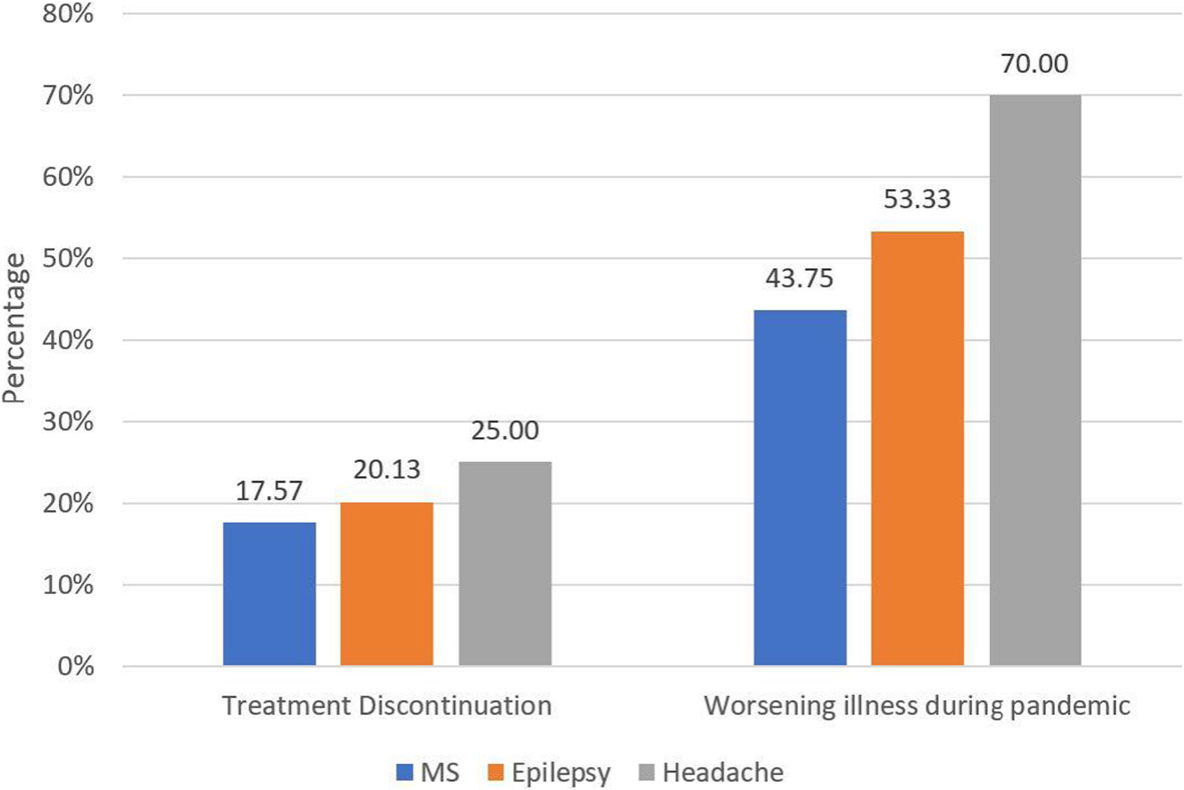
Impact of the pandemic on three common neurological illnesses in Jordan. Patients with headache were most likely to report discontinuation of medication or worsening of their illness since the start of the pandemic, followed by epilepsy patients and patients with MS, respectively

Figure 1 demonstrates the frequency of reporting treatment discontinuation and worsening clinical course since the start of the pandemic in the three common neurological illnesses described in previous paragraphs.

## Discussion

In this survey, most respondents had positive attitudes towards the COVID-19 pandemic. At the same time, the negative impacts of the pandemic on patients with neurological illnesses in Jordan were evident. Understanding the attitudes and beliefs of a population towards the pandemic has significant implications for implementing and planning of mitigation strategies and for vaccination campaigns. We found that most patients (96%) who participated in the survey believed in the seriousness of the pandemic and demonstrated a positive attitude towards it such as adherence to prevention measures and support of national infection control plans. A previous national survey in April of 2020 revealed similar findings [14]. Therefore, despite different methodologies and target populations, the findings of our study reflect that awareness and positive attitude towards the pandemic among Jordanians remain high. We also found that nearly 97% of patients resorted to the internet and media outlets for medical information about the pandemic, a critical finding in an era where public perceptions about the pandemic are increasingly shaped by social media [15].

The findings of this study also reveal some aspects of how COVID-19 pandemic impacted health services in Jordan. Nearly one in five clinic visitors had their appointments delayed due to the interruptions of health services caused by the pandemic. Also, a similar percentage of patients with MS, epilepsy, and migraine or tension headache reported medication interruptions because of the pandemic and quarantine measures. On the other hand, nearly one in three patients with epilepsy or headache reported sleep disturbances, and half of those believed the pandemic was responsible. This is significant since sleep disturbance is a known trigger in both epilepsy and migraine headache [16, 17]. Finally, nearly one in two of surveyed patients reported new events or worsening illnesses since the start of the pandemic. This finding mostly is the outcome of a multitude of factors, such as medication interruptions, sleep disturbances, new social and financial stressors, limitations on accessibility to health services, pandemic-related anxiety, lifestyle changes, and others [18].

Surveys from other countries and regions reported varying but consistent findings underpinning the impacts of the COVID-19 pandemic. For instance, surveys that have been published recently reported that between 4 and 35% of persons with epilepsy had seizure worsening during the pandemic, and the worsening was mainly correlated with epilepsy severity, sleep disturbances, and COVID-19-related factors [16, 18]. Also, in a UK-based study that surveyed persons with epilepsy, a third reported difficulty accessing medical services, with 8% having had an appointment canceled. Meanwhile, medication shortages were noted by approximately 30% of neurologists in a survey of the American Epilepsy Society members [19]. On the other hand, a survey of 176 MS patients from Saudi Arabia found that 15% of the patients had a relapse but did not seek medical help because of the pandemic, while 15.9% stopped their DMTs, and 35.2% reported missing drug infusions or refills [20]. Moreover, in a survey of 1018 persons with migraine headache from Kuwait, 59.6% of sampled patients reported an increase in migraine frequency, and 78.1% reported sleep disturbances [17].

This study is not without limitations. Although recruiting patients from outpatient clinics reduced selection bias as opposed to online surveys, it remains based on a single center and medical diagnoses were ascertained retrospectively from patient files. Additionally, the establishment of a direct causal relationship through observational surveys is difficult. Nonetheless, this study is a steppingstone for future efforts in this regard.

## Conclusion

The impacts of COVID-19 pandemic on neurology patients in Jordan are diverse and evident across the spectrum of neurological illnesses. More studies are necessary to further delineate the impacts of pandemic on other aspects of health services in the country and, importantly, to draw appropriate conclusions for the future.

## Data Availability

Available from Mohammad Athamneh upon reasonable request.

## Abbreviations

SARS: Severe acute respiratory syndrome
COVID-19: Coronavirus disease of 2019
SARS-CoV-2: Severe acute respiratory syndrome coronavirus 2
MS: Multiple sclerosis
DMT: Disease-modifying treatment
ASM: Anti-seizure medication
